# Scientometric analysis of chemotherapy of canine leishmaniasis (2000–2020)

**DOI:** 10.1186/s13071-020-04544-x

**Published:** 2021-01-09

**Authors:** A. I. Olías-Molero, E. Fontán-Matilla, M. Cuquerella, J. M. Alunda

**Affiliations:** 1 Department of Animal Health, Faculty of Veterinary Medicine, University Complutense Madrid, 28040 Madrid, Spain; 2 Instituto de Investigación Hospital 12 de Octubre, Avda. Andalucía s/n, 28041 Madrid, Spain

**Keywords:** Allopurinol, Amphotericin, Antimonials, Canine leishmaniasis, Chemotherapy, *Leishmania* spp., *Leishmania infantum*, Miltefosine, Sb^V^

## Abstract

**Background:**

Zoonotic visceral leishmaniasis by *Leishmania infantum* is a first-order pathology in canine veterinary clinics in endemic areas. Moreover, canine infections are considered the main reservoir for human disease; despite their importance in the control of the disease within a One Health approach, no scientometric study has been published. Aims of the study included analyzing the impact of canine leishmaniasis (CanL) on the scientific literature, drugs or combinations used, trends in the period from 2000 to 2020 and efficacy criteria employed.

**Methods:**

A Web of Science (WOS)-based analysis of publications on CanL and chemotherapy of the disease in the period 2000–2020 was carried out using a stepwise methodology. Data were analyzed by year, geographical origin, chemical groups, drugs and combinations, and efficacy criteria.

**Results:**

Reports on CanL (*n* = 3324) represented < 16% of all publications on leishmaniasis (*n* = 20,968), and of these around 18% (*n* = 596) were related to chemotherapy. Publication records on CanL followed the distribution of the infection by *L. infantum* in endemic areas although Mediterranean countries were overrepresented in the reports on chemotherapy of CanL. Publications on the main antileishmanial drugs used in clinical practice showed a sustained tendency in the period analyzed. Pentavalent antimonials (Sb^V^), alone or in combination with allopurinol, represented > 50% of all publications on chemotherapy of CanL despite the availability of more recently marketed drugs.

**Conclusions:**

Chemotherapy of CanL still relies on Sb^V^ and combinations and to a lesser extent on miltefosine (MIL). Reports on chemotherapy are scarce and mostly publicly funded, and the variability of experimental conditions hampers the direct comparison of the efficacy of drugs, combinations and schedules. The vast majority of reports on efficacy do not include any information on supportive therapy; this reduces the actual value of the studies if intended for the practical management of the disease. Complete reports on the chemotherapy (etiological + symptomatic) would add value to the trials performed.
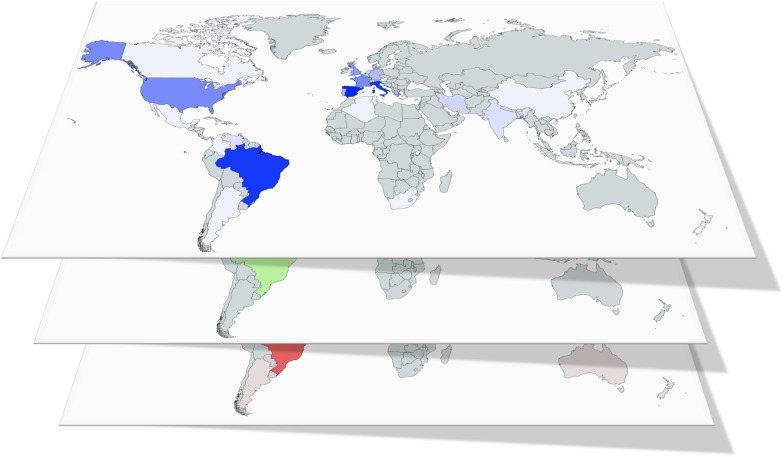

## Background

Leishmaniasis is a widely distributed group of vector-borne parasitic diseases caused by kinetoplastids from the genus *Leishmania*. Clinical course relates to the *Leishmania* spp. involved and the functionality of the immune system of the hosts. Visceral leishmaniasis (VL), provoked by *Leishmania donovani* and *Leishmania infantum*, is the most severe condition; it is prevalent in Asia, South America and southern Europe [1–3], and its expansion to northern latitudes has been reported [4, 5]. Moreover, new transmission patterns have been identified, linked to immunocompromised patients, non-vectorial transmission and solid organ transplant recipients [6, 7]. Dogs can be infected by several *Leishmania* spp. [8] and are considered the main reservoir for human infections by *L. infantum* [9]. Canine leishmaniasis (CanL) is very frequent, reaching > 30% prevalence in some “hot spots” [10], and constitutes a first-order veterinary pathology in endemic areas. Control of CanL has important shortcomings since marketed vaccines have limitations [11, 12], and environmental control is infeasible. Culling of infected dogs is debatable, and its actual impact has been challenged [13–15]. Therefore, control of CanL largely relies on the use of repellents, and mainly on chemotherapy of infected dogs. The therapeutic arsenal includes the same compounds used for the treatment of human leishmaniasis: antimonials (Sb^V^), amphotericin (AmB), miltefosine (MIL), allopurinol and paromomycin, among other drugs and combinations [16–18]. Treatment schedules are highly variable among veterinarians, and some efforts to harmonize chemotherapy schemes have been made [19, 20]; for the time being, variability is the rule.

Despite the importance of CanL by *L. infantum* in both dog clinics and for public health because of its zoonotic transmission—a clear example of the need for the One Health approach—few scientometric contributions on leishmaniasis are available, and none have focused on CanL [21–23]. Therefore, the aim of our study was the analysis of anti-leishmanial drugs employed in the treatment of CanL, using Web of Science (WOS) as the data source, in the twenty-first century. The time window (2000–2020) was selected because of the growing awareness of the need to control the infection in the main reservoir, the launch of MIL, the only available antileishmanial oral drug, and the publication of several guidelines for CanL chemotherapy.

## Methods

### Documents and selection criteria

Analysis followed a stepwise methodology [24] using the WOS Core collection as data source. Documents containing the terms “leishmaniosis” or “leishmaniasis” in their titles, abstracts or key words were selected. Reports were further restricted to CanL (Leishmaniosis OR leishmaniasis + dogs OR canine) and, finally, those directly focused on chemotherapy (leishmaniosis OR leishmaniasis + dogs OR canine + treatment OR therapy). Data obtained were screened for chemotherapeutic agents (leishmaniosis OR leishmaniasis + dogs OR canine + Drugs). Initially, 33 drugs were searched (i.e. allopurinol, meglumine, meglumine antimoniate, glucantime, antimony, Sb^III^, Sb^V^, pentostam, amphotericin, amphotericin B, aminosidine, paromomycin, MIL, pentamidine, domperidone, metronidazole, ketoconazole, fexinidazole, marbofloxacin, thymol, eugenol, furazolidone, sesquisterpene, fluconazole, itraconazole, bisabolol, silybin, dehydrosilybin, dehydroisosilybin, allicin, resazurin, sitamaquine). No results were obtained for the last seven items.

Publications obtained were grouped: [(leishmaniosis OR leishmaniasis) + (Dogs OR canine) + (meglumine OR meglumine antimoniate OR antimony OR glucantime OR Sb^V^ OR Sb^III^ OR pentostam)]: *antimonials*; [(leishmaniosis OR leishmaniasis) + (dogs OR canine) + (amphotericin OR amphotericin B)]: *amphotericin B*; [(leishmaniosis OR leishmaniasis) + (dogs OR canine) + (miltefosine or milteforan)]: *miltefosine*; [(leishmaniosis OR leishmaniasis) + (dogs OR canine) + (aminosidine OR paromomycin)]: *paromomycin*; [(leishmaniosis OR leishmaniasis) + (dogs OR canine) + (metronidazole OR ketoconazole OR fexinidazole OR itraconazole OR fluconazole]): *azoles*; [(Leishmaniosis OR Leishmaniasis) + (Dogs OR canine) AND (thymol OR eugenol OR sesquiterpene)]: *essential vegetable oils*; [(leishmaniosis OR leishmaniasis) + (dogs OR canine) AND (pentamidine OR pentamidina)]: *pentamidine*. Results obtained were analyzed considering year of publication; geographical origin (i.e. senior author’s affiliation); drugs, combinations and treatment schedule when available; efficacy criteria: improvement of clinical status and lesions, normalization of biochemical and hematological parameters; reduction of parasite burden; reduction of specific antibodies.

### Analysis of data

Comparison of series (documents/year) was carried out with normalized values (z) using the formula:

$$ Z = \frac{x - \mu }{\sigma }, $$

where *x* is the individual value, *μ* is the mean value of the series, and *σ* is the standard deviation of the series. Tendencies of the number of documents related to the identified drugs or groups of drugs throughout the sampled period were calculated with the polynomial function:

$$ f\left( x \right) = a_{n} *x_{n} + \cdots + a_{3} *x_{3} + a_{2} *x_{2} + a_{1} *x + a_{0} , $$

where *a*_0_⋯*a*_*n*_ are constants, and n is the polynomial order. Data on dog populations and dog householders were obtained from http://www.worldatlas.com, http://www.statista.com and http://www.moag.gov.il. No data on the Iranian dog population could be obtained. Analysis of results and figures were carried out with GraphPad Prism 6.0. Level of significance was set at *P* < 0.05. Maps were elaborated with mapchart.net.

## Results

### Pattern of publications on CanL: growing, aggregated and related to the distribution of the disease, human population, dog numbers and social awareness

**Fig. 1:**
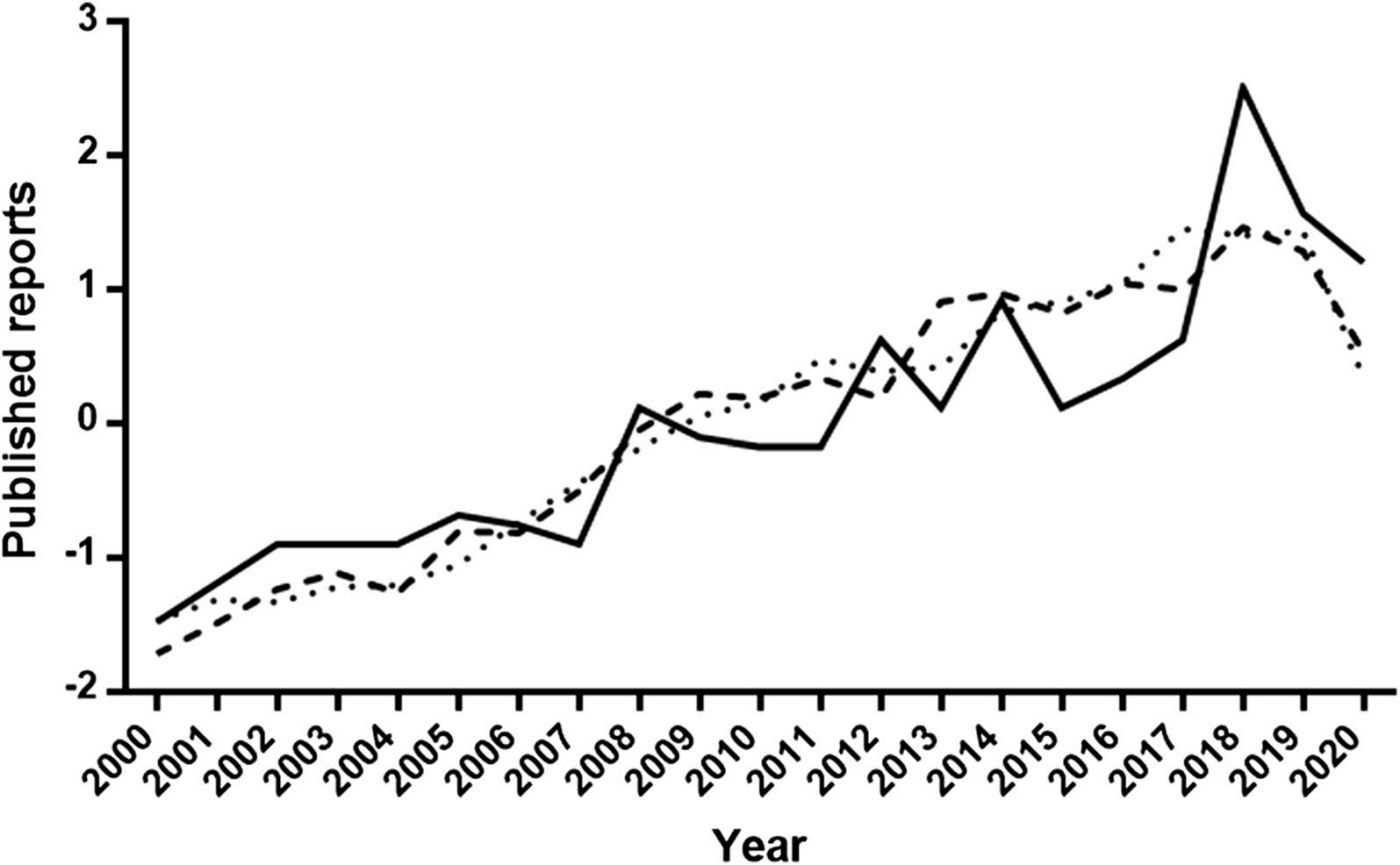
Evolution of articles recovered from WOS in the period 2000–2020. Values given are normalized numbers of publications on leishmaniasis (….), canine leishmaniasis, CanL (- - -) and therapeutics of canine leishmaniasis (__).

WOS covered articles/documents on leishmaniasis between 2000 and 2020 reached a total number of 20,968. Less than 16% (3324), mostly written in English (> 90%), corresponded to CanL, with 596 documents including the terms “treatment” or “therapy.” Papers related to CanL displayed an increase in the analyzed period although the pattern of contributions on chemotherapy showed higher year-to-year variations (Fig. 1); variability is probably related to the significantly lower value compared to those from leishmaniasis and CanL.

**Fig. 2:**
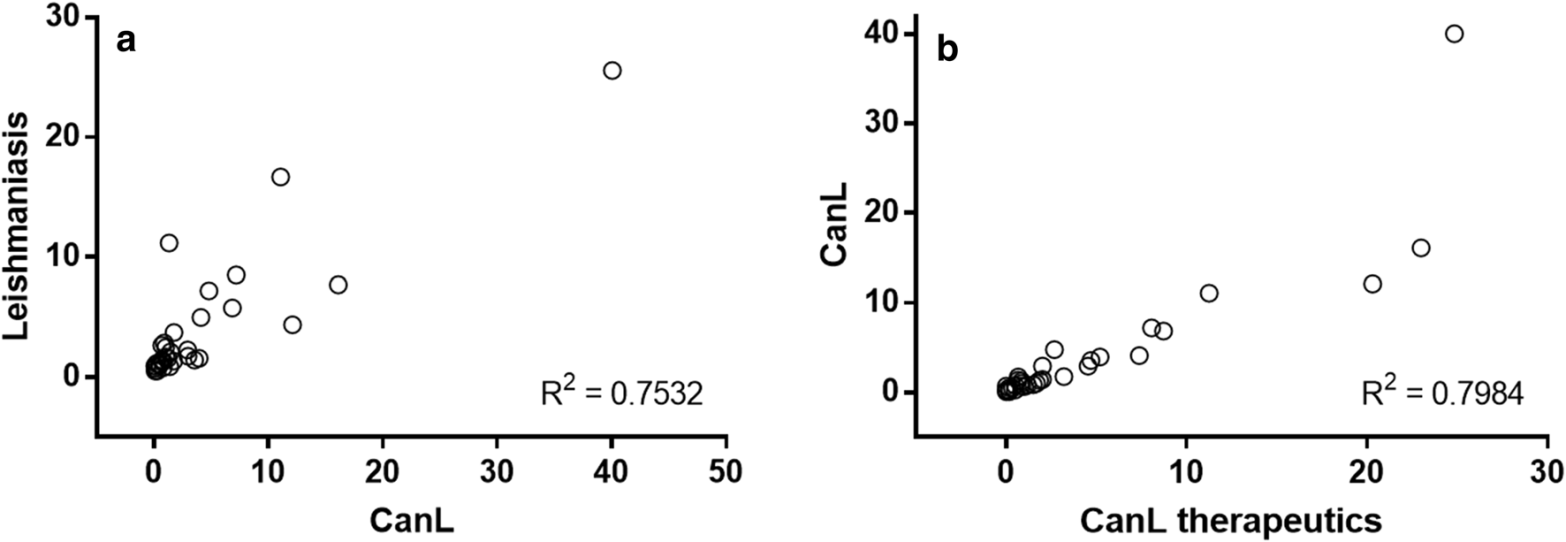
Relationship between publications on leishmaniasis and canine leishmaniasis (**a**) and canine leishmaniasis and chemotherapy of the dog infection (**b**). Each dot corresponds to a country.

Analysis of affiliations showed that 94 countries out of 112 with published articles on leishmaniasis were the origin of papers on CanL. Considering the countries producing > 100 papers in the analyzed period, distribution showed an overdispersed pattern; hence, only three countries represented > 65% of all papers, with Brazil being the most productive (1331) with 40.04% of the articles published in the studied period (Additional file 1: Table S1). These countries were also those with a higher representation when the therapeutics of CanL were considered although the values were lower and similar among them (Italy: 20.30%; Spain: 22.99%; Brazil: 24.83%). For comparative purposes, data from “leishmaniasis” were analyzed in a similar way; the number of publications was also aggregated. Scientific output on leishmaniasis and CanL and between CanL and chemotherapy of the dog infection was correlated (*R*^*2*^ = 0.7532, *P* < 0.0001 and *R*^*2*^ = 0.7984, *P*<0.0001, respectively) (Fig. 2). Despite the correlation, there were outliers in both cases. Thus, USA and India with 3500 and 2344 papers on leishmaniasis, respectively, only had a modest CanL representation (11.07% and 1.32%, respectively) (Additional file 1: Table S1). Russia was not present among the most productive countries, and China had figures comparable to those of Peru or Sudan + South Sudan (Additional file 1: Table S1). Given the zoonotic transmission of CanL by *L. infantum*, the relationship between scientific output on CanL and the human and dog population from the most represented countries was examined. No clear correlation emerged with the human or dog population from the countries (*R*^*2*^ < 0.2). However, excluding non-endemic areas for CanL (India, Japan, Australia, the USA, Canada) and countries without an available canine census, the scientific production on CanL from each country was significantly correlated to its human (*R*^*2*^ = 0.7465, *P* *=* 0.0001) and dog (*R*^*2*^ = 0.8793, *P* < 0.0001) population.

### Geographical distribution of drug reports is overdispersed

**Fig. 3:**
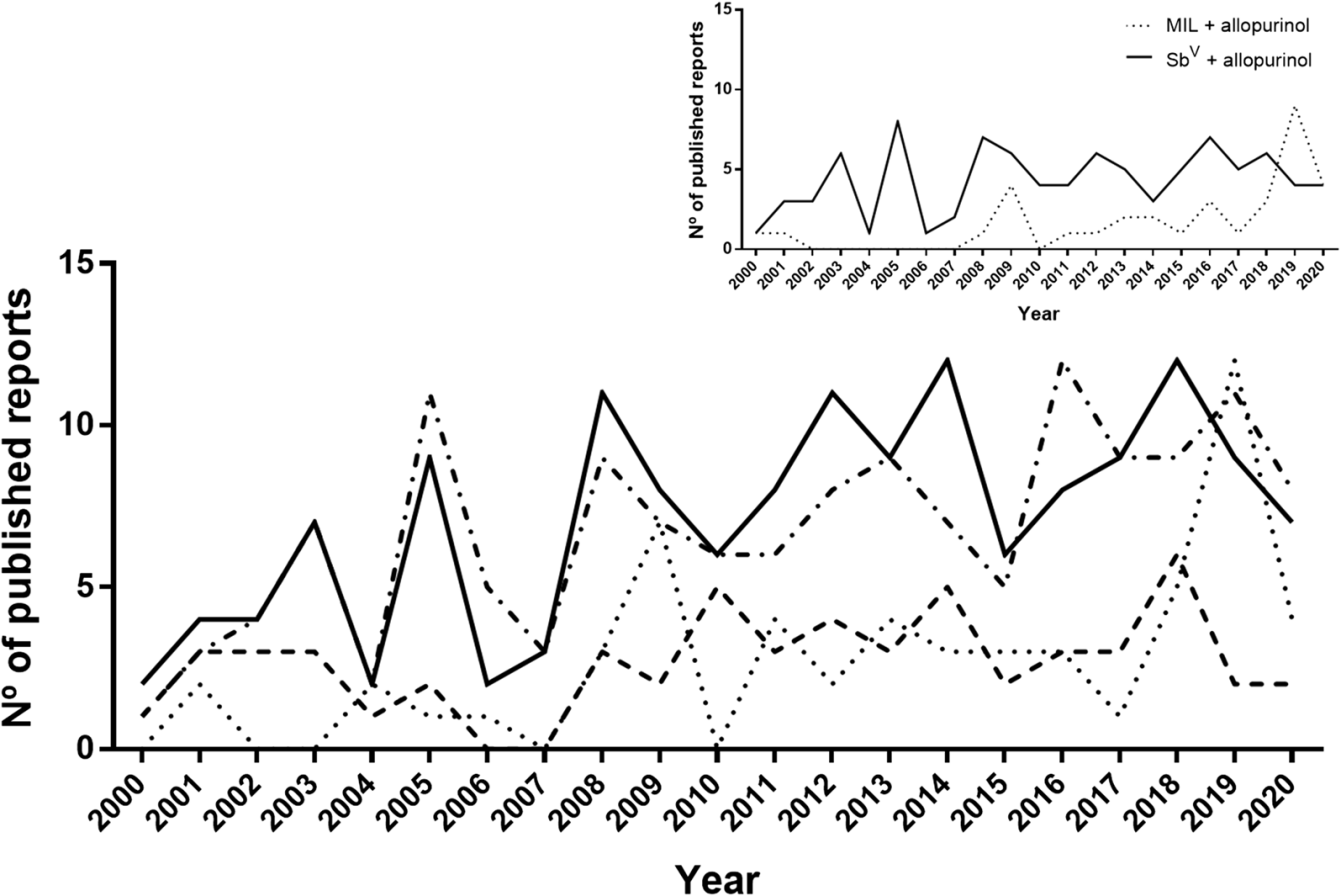
Temporal evolution (2000–2020) of WOS covered published reports on the main antileishmanial drugs used in veterinary clinics. Allopurinol (-.-.- ); amphotericin (- - -); antimonials (Sb^V^) (___); miltefosine (…….). Insert. Number of articles on the combination of Sb^V^ + allopurinol and miltefosine + allopurinol throughout the study period.

Individual analysis of the 596 publications recovered in WOS on chemotherapy of CanL showed that in 461 of them one or more of the selected drugs were included (see Material and methods). Presence of paromomycin was scarce (15 reports throughout the period), and > 60% of all publications were related to Sb^V^ and allopurinol. These drugs, along with AmB and MIL, with 56 and 57 papers, respectively, were the most represented. Reports with Sb^V^ and allopurinol were, in general, consistently the most frequently published in the examined period with a steady increase up to the year 2011. MIL and AmB, although with lower numbers, displayed a similar trend except for the year 2019 with a high number of publications (*n* = 12) on MIL (Fig. 3). Presence of allopurinol was related to its combination with either Sb^V^ (*n* = 91) or MIL (*n* = 34) (insert in Fig. 3) more than to the use of this drug as monotherapy. In fact, the number of publications on drugs and combinations throughout the period evaluated showed comparable patterns.

Scientific output on the selected drugs was highly aggregated considering the geographical origin of the publications. Reports came from fewer than 30 countries (Sb^V^: 26; AmB: 22; MIL: 14; allopurinol: 21; Sb^V^ + allopurinol: 17; MIL+ allopurinol: 11). Moreover, only two countries, Italy and Spain, originated > 50% of reports published with all drugs and combinations considered (Additional file 2: Table S2). Sb^V^ was more frequently reported than MIL, particularly in the countries with a higher number of articles (2.3- to 3.6-fold). Similarly, the combination of allopurinol + Sb^V^ was, as median value, two times more frequent than allopurinol + MIL. No reports on MIL were found for Israel, and AmB reached some significant figures in Brazil (*n* = 14) and Spain (*n* = 12).

Most research published on CanL corresponded to public institutions (i.e., universities, research institutions, foundations) irrespective of the country considered. In 50% of papers on CanL there was a formal recognition of the research funding (1627 out of 3324). The vast majority of publications on CanL were funded by public institutions whereas only 84 publications (4.8%) acknowledged private support. Considering the papers on chemotherapy, the participation of Pharma funding increased, reaching 10.67% for Sb^V^ and 20% for MIL.

### Reports on the efficacy of anti-CanL drugs and combinations: scarce and highly variable

Considering the evaluation of antileishmanial efficacy, only 122 publications out of 596 dealing with chemotherapy or treatment of CanL fit the objective in the sampled period. All other reports corresponded to reviews, immunotherapy, heat-inducing radiofrequencies or scarcely employed drugs and were excluded from the analysis. Sb^V^, alone or in combination with allopurinol, represented > 50% of all reports on chemotherapy of CanL. In addition, in 21% of all papers included in this section, antimonials were the only antileishmanial agent administered. Allopurinol was employed alone (20% of papers) or in combined therapy, particularly with Sb^V^ (*ca.* 33% of the publications). For its part, MIL, administered alone or in combination with allopurinol, was reported by 19% of publications.

Publications on the therapeutics of CanL were largely related to *L. infantum* (= *L. chagasi*) with only four reports on other *Leishmania* spp. [*L. major*, *L. tropica* and *L. *(*Viannia*)* braziliensis*]. Analysis of the selected papers showed that there was great variability among them considering the efficacy criteria, methods and timing of analyses, type of biological sample, dog breed and physiological status, or type of infection (natural vs. experimental) among other factors. Improvement of the clinical condition of the animals and reduction or disappearance of lesions were predominant (108 and 31 papers, respectively) followed by the normalization of hematological and biochemical parameters (e.g. plasma protein levels, renal function markers). However, more specific parameters such as the specific antibody response and parasite burden were less represented. Hence, antibody response, determined by IFAT or ELISA, was only considered in 12 papers and the evaluation of parasite burden, by counting or qPCR, in 10 articles. This shows that many of the reports WOS covered were related to clinical cases, trials with very low numbers of dogs or clinical observations in studies not directly focused on CanL. Treatment with antimonials reduced/eliminated clinical signs and, when determined, elicited a reduction of anti-*Leishmania* antibody levels. The combination of Sb^V^ with allopurinol elicited comparable results and was considered the most efficacious treatment against CanL. Allopurinol reportedly reduced the clinical signs, and, in most cases, a reduction of specific antibodies was observed. However, its inferior chemotherapeutic value, administered alone, compared to combinations with MIL or Sb^V^ was highlighted. MIL, alone or in combination with allopurinol, was efficacious in reducing signs and improving the clinical condition of treated dogs; results on its effect on antibodies were variable or not reported. Results with AmB were positive, with the disappearance of clinical signs and reduction of parasite burden. The number of publications related to other drugs was scarce, although marbofloxacin, aminosidine, pentamidine and oleilphosphocholine were reported to be efficacious, whereas metronidazole, combined with spiramicine or enrofloxacin, was not; difloxacin + metronidazole reduced the clinical signs and diminished the antibody levels. Several papers involved the use of immunomodulators (e.g. domperidone, magnesium ammonium phospholinoleate anhydride). It is noteworthy to indicate that as a rule no supportive therapies, besides the etiological treatment, were indicated in the published reports.

There was a wide variation in the timing of post-treatment assessment of efficacy, ranging from 28 days to over 1 year. Similarly, experimental designs included for the most part naturally infected dogs with different initial clinical conditions (only five papers dealt with experimentally infected dogs). Moreover, efficacy was tested in different dog breeds (e.g. mongrels, beagles, boxers, retrievers) particularly in the cases of multicentric trials with larger numbers of dogs.

## Discussion

The aggregation of scientific contributions on CanL found in endemic countries is evidence of the predominance of *L. infantum* as the causative agent and the public health relevance of this zoonotic infection. This was supported by the strong correlation found in endemic areas between the number of publications on CanL and the human and dog populations. This was particularly clear in Mediterranean countries with low values of the ratio between the number of reports on leishmaniasis and CanL (e.g. Italy: 2.29; Greece: 2.54). This low ratio is probably due to the relatively low impact of the human disease in the region [25, 26] and the high prevalence of CanL [27–29]. Similarly, endemicity of the disease and the number of dogs in Brazil (58% of households) [30] could account for its highest participation (*ca.* 40% of all papers on CanL). The high value of the ratio found for India (53) relates to the minimal role of dogs in the mainly non-zoonotic transmission of *L. donovani* whereas no easy explanation was found for the reduced scientific output from China, where *L. infantum* infection is endemic. For its part, the presence of both leishmaniasis and CanL in the USA, with modest numbers of domestic human and canine cases, possibly reflects their scientific and global interest. Publications on CanL and chemotherapy of CanL were correlated despite the comparatively lower chemotherapy values in Brazil. Predominance of endemic countries in the publication record on chemotherapy for CanL was expected. Drug availability, besides the social and veterinary awareness related to the disease, could explain the overrepresentation of Mediterranean countries (Italy and Spain combined: 45% of all papers).

MIL, a repurposed anti-tumor drug, was approved by the European Medicines Agency in 2002 for the treatment of human VL [31] and has been marketed in Europe for veterinary use since 2007. Oral administration and the good results reported [32–34] suggest a higher presence in the scientific literature. However, despite > 10 years on the market, papers on the alkylphospholipid represented < 20% of the total, *ca.* one third of those including antimonials, and there were no published records in 2010. Whether or not the comparatively easy induction of resistance in vitro [35] and reports on its lower efficacy [36] are responsible for the comparatively low representation of MIL in the literature should be analyzed. Moreover, the late introduction of MIL to the Brazilian market (Milteforan® was approved in 2016), together with active policies in favor of culling infected dogs in this country [37], could account for this relative scarcity of scientific reports on MIL.

The degree of aggregation with the other drugs was comparable although there were some striking findings. Reports of AmB in Brazil and the USA are understandable given the late approval of veterinary presentations of MIL but the figures on the antibiotic from countries with a wide range of antileishmanial drugs against CanL (e.g. France, Spain) deserve deeper study. Antimonials have been, and still are in most regions, the standard etiological treatment of CanL despite the conflicting reports on the toxicity of Sb^V^ [16, 17, 38, 39], incomplete knowledge of its mechanism of action (MoA) [40] and reports on the emergence of resistance phenomena in some canine isolates of *L. infantum* [41]. Over 50% of all publications on the chemotherapy of CanL in the period dealt with the combination of Sb^V^ with allopurinol. Use of this combined therapy has been growing, and new formulations have been tested [42–44]. No clear conclusion can be drawn from the results obtained on the treatment of CanL with Sb^V^, alone (e.g. Brazil) or in combination with allopurinol (e.g. Spain and Italy). Further analyses considering the variation of *L. infantum* strain sensitivity, market issues, perception of veterinary clinicians, funding and the role of veterinary associations, among other aspects, could clarify these regional differences.

Heterogeneity of designs (natural vs. experimental infections, dog breeds, follow-up, parameters determined and timing of analyses) precludes the accurate comparison of drugs, combinations and schedules, and a meta-analysis is required. However, based on the publications analyzed in our study, the combination of Sb^V^ (100 mg/kg/day, subcutaneous), in a single dose or divided (50 + 50 mg/kg), for 28–30 consecutive days, followed by the long-term (> 6 months) daily administration of allopurinol (5–10 mg/kg/day), seems to reduce clinical signs and lesions, biopathological alterations, parasite burdens in target organs and plasma levels of specific anti-*Leishmania* antibodies. Hence, this combination could be considered the first treatment choice in CanL. Combination of MIL (2 mg/kg/day, 28 days) + long-term administration of allopurinol (5–10 mg/kg) elicits significant clinical and biopathological improvements of treated animals besides reduction of parasite burden. However, relapses tend to be more frequent in this case compared to the Sb^V^ + allopurinol combination. Moreover, medication with MIL does not reduce the levels of specific antibodies, thus complicating the follow-up. On these grounds, MIL+ allopurinol could be considered a second-line treatment. These results obtained in the scientometric analysis carried out seem to support the published recommendations on the management of CanL [19, 20, 45].

AmB is an excellent leishmanicidal drug, and lower toxicity formulations (e.g. liposomes) are the first choice treatment for human visceral leishmaniasis [46, 47]. Therefore, its use in dog leishmaniasis is not recommended in order to minimize cross resistance between canine and human isolates of *L. infantum*. The main MoA of AmB, interference with ergosterol biosynthesis in *Leishmania*, probably makes the emergence of resistance to this antibiotic a rare event, and it is widely used in human medicine as a systemic antifungal [48]. However, resistant strains have been produced under laboratory conditions, and several reports on resistance in humans have been published [18, 49]. Despite this, at least when bibliographic production is considered, AmB is used against CanL, and no tendency toward its reduction has been observed in the time window analyzed. Therefore, the caveat against the medication of dogs with AmB seems reasonable when no new drugs are foreseen. No available drug or drug combination against CanL produces the parasitological cure [17], relapses are frequent, and the potential for development of resistant isolates of *L. infantum* is present [37]. Resistance of *Leishmania* to the currently used drugs has been widely reported [50], and there are indications of reduced sensitivity of *L. infantum* after repeated treatment with Sb^V^ in both humans [51] and dogs [52]. Moreover, it has been shown that resistance to allopurinol can be induced in dog isolates of *L. infantum* [53]. Consideration of the possible cross resistance (dogs and human isolates) and zoonotic nature of CanL indicates the need to use different chemical families to treat human and CanL should be considered.

## Conclusions

We are aware of the limitations of this overview including the need to refine the search terms and the limitations of WOS as a data source. However, our analysis clearly shows that publications on CanL are largely focused on *L. infantum* infections in European endemic areas and Brazil. This predominance is probably related to the high prevalence of canine infections and resource allocation. Reports on chemotherapy of the canine disease are comparatively scarce, and the variety of conditions (e.g., initial clinical condition of the animals, dog breed and age, *Leishmania* isolate, parameters employed in the follow-up) precludes direct comparison among them; this suggests the importance of a meta-analytic study. Despite variations, combined therapy of Sb^V^ + long-term treatment with allopurinol seems to be the best therapeutic option. The number of published reports receiving private funding is surprisingly low considering the obvious interest for Pharma companies. Whether or not the scientometric findings reflect the actual use of the drugs in veterinary clinics is not known. Notably, the majority of publications on chemotherapy only included antileishmanial agents. However, unless administration of supportive therapy is clearly indicated, the value of these studies is limited and far from the prevailing practical conditions. We strongly advocate for a complete account of all medications given to experimental dogs to add value to the assays performed. Our analysis has no market considerations, but high-content trials (complete information, standard parameters determined) would be more helpful and would allow a fair comparison of efficacy. The use of the same drugs against *L. infantum* infections in human and veterinary medicine requires careful management to reduce the risk of selection of drug-resistant isolates.

## Supplementary Information

**Additional file 1: Table S1.** Scientific output on leishmaniasis, canine leishmaniasis (CanL) and chemotherapy of CanL, by country of origin, recovered by WOS in the period 2000–2020*.

**Additional file 2: Table S2.** Scientific output in some selected countries of frequently used drugs and combinations against CanL covered by WOS in the period 2000–2020*.

## Data Availability

Data and materials are available from authors.
