# Supplementary material for: Scientometric analysis of chemotherapy of canine leishmaniasis (2000–2020)
Source: Parasit Vectors. 2021 Jan 9;14:36. doi: 10.1186/s13071-020-04544-x (PMC7796616; doi:10.1186/s13071-020-04544-x)
Supplement: Supplementary file 1 — Additional file 1: Table S1. Scientific output on leishmaniasis, canine leishmaniasis (CanL) and chemotherapy of CanL, by country of origin, recovered by WOS in the period 2000–2020*. [file 13071_2020_4544_MOESM1_ESM.docx]

**Additional Information**

**Table S1**. Scientific output on leishmaniasis, canine leishmaniasis (CanL) and chemotherapy of CanL, by country of origin, recovered by WOS in the period 2000-2020.*

| **Country** | **Leishmaniasis** | **CanL** | **Chemotherapy CanL** |
| --- | --- | --- | --- |
| BRAZIL | 5365 (25.59) | 1331 (40.04) | 148 (24.83) |
| USA | 3500 (16.69) | 368 (11.07) | 67 (11.24) |
| INDIA | 2344 (11.18) | 44 (1.32) | 11 (1.85) |
| UNITED KINGDOM | 1789 (8.53) | 239 (7.19) | 48 (8.05) |
| SPAIN | 1613 (7.69) | 535 (16.10) | 137 (22.99) |
| IRAN | 1509 (7.20) | 159 (4.78) | 16 (2.68) |
| FRANCE | 1211 (5.78) | 227 (6.83) | 52 (8.72) |
| GERMANY | 1044 (4.98) | 136 (4.09) | 44 (7.38) |
| ITALY | 921 (4.39) | 402 (12.09) | 121 (20.30) |
| SWITZERLAND | 784 (3.74) | 58 (1.74) | 19 (3.19) |
| BELGIUM | 598 (2.85) | 29 (0.87) | 9 (1.51) |
| CANADA | 556 (2.65) | 22 (0.66) | 6 (1.01) |
| COLOMBIA | 517 (2.47) | 33 (0.99) | 5 (0.84) |
| TURKEY | 475 (2.27) | 97 (2.92) | 12 (2.01) |
| NETHERLANDS | 441 (2.10) | 48 (1.44) | 12 (2.01) |
| ISRAEL | 368 (1.76) | 98 (2.95) | 27 (4.53) |
| AUSTRALIA | 352 (1.68) | 34 (1.02) | 10 (1.68) |
| PORTUGAL | 334 (1.59) | 131 (3.94) | 31 (5.20) |
| TUNISIA | 334 (1.59) | 41 (1.23) | 5 (0.84) |
| JAPAN | 299 (1.43) | 22 (0.66) | 5 (0.84) |
| GREECE | 297 (1.42) | 118 (3.55) | 28 (4.70) |
| ETHIOPIA | 283 (1.35) | 17 (0.51) | 1 (0.17) |
| VENEZUELA | 283 (1.35) | 27 (0.81) | 7 (1.17) |
| ARGENTINA | 279 (1.33) | 57 (1.71) | 4 (0.67) |
| MEXICO | 267 (1.27) | 23 (0.69) | 0 (0.00) |
| PAKISTAN | 241 (1.15) | 7 (0.21) | 0 (0.00) |
| SUDAN + SOUTH SUDAN | 229 (1.09) | 17 (0.51) | 1 (0.17) |
| PEOPLE’S R. CHINA | 220 (1.05) | 22 (0.66) | 7 (1.17) |
| PERU | 209 (1.00) | 15 (0.45) | 2 (0.34) |
| NEPAL | 200 (0.95) | 2 (0.06) | 1 (0.17) |
| SAUDI ARABIA | 192 (0.92) | 6 (0.18) | 0 (0.00) |
| BANGLADESH | 187 (0.89) | 7 (0.21) | 0 (0.00) |
| CZECH REPUBLIC | 187 (0.89) | 46 (1.38) | 4 (0.67) |
| SWEDEN | 187 (0.89) | 8 (0.24) | 1 (0.17) |
| MOROCCO | 168 (0.80) | 26 (0.78) | 3 (0.50) |
| EGYPT | 154 (0.73) | 3 (0.09) | 0 (0.00) |
| THAILAND | 114 (0.54) | 9 (0.27) | 3 (0.50) |
| SRI LANKA | 110 (0.52) | 3 (0.09) | 0 (0.00) |
| KENYA | 108 (0.52) | 1 (0.03) | 0 (0.00) |

*Numbers correspond to the number of publications. In brackets, the % of the total output within each column: Leishmaniasis (n= 20968); CanL (n=3324); chemotherapy of CanL (n=596).
