# Supplementary material for: Scientometric analysis of chemotherapy of canine leishmaniasis (2000–2020)
Source: Parasit Vectors. 2021 Jan 9;14:36. doi: 10.1186/s13071-020-04544-x (PMC7796616; doi:10.1186/s13071-020-04544-x)
Supplement: Supplementary file 2 — Additional file 2: Table S2. Scientific output in some selected countries of frequently used drugs and combinations against CanL covered by WOS in the period 2000–2020*. [file 13071_2020_4544_MOESM2_ESM.docx]

**Additional Information**

**s**

| **Countries** | **Drugs and combinations** | | | | | |
| --- | --- | --- | --- | --- | --- | --- |
|  | **Allopurinol** | **AmB** | **Sb^V^** | **MIL** | **Sb^V^ + Allopurinol** | **MIL + Allopurinol** |
| Brazil | 14 | 14 | 26 | 10 | 7 | 4 |
| Spain | 43 | 12 | 43 | 12 | 33 | 9 |
| Italy | 40 | 5 | 37 | 16 | 26 | 13 |
| USA | 10 | 5 | 12 | 3 | 7 | 2 |
| France | 13 | 7 | 23 | 7 | 12 | 2 |
| United Kingdom | 11 | 7 | 9 | 7 | 3 | 4 |
| Germany | 11 | 2 | 6 | 5 | 6 | 3 |
| Portugal | 8 | 4 | 12 | 4 | 8 | 3 |
| Greece | 13 | 3 | 12 | 4 | 10 | 3 |
| Israel | 14 | 3 | 7 | 0 | 6 | 0 |
| Switzerland | 7 | 3 | 3 | 2 | 3 | 2 |
| Iran | 0 | 2 | 4 | 1 | 0 | 0 |
| Turkey | 2 | 1 | 0 | 0 | 1 | 0 |
| The Netherlands | 1 | 2 | 1 | 0 | 1 | 0 |
| India | 1 | 1 | 3 | 0 | 1 | 0 |
| Australia | 0 | 1 | 1 | 0 | 0 | 0 |
| Belgium | 3 | 1 | 3 | 1 | 2 | 1 |
| Venezuela | 0 | 0 | 0 | 2 | 0 | 0 |
| Colombia | 1 | 1 | 3 | 0 | 1 | 0 |
| Argentina | 0 | 0 | 1 | 0 | 0 | 0 |

*Numbers correspond to the number of published records.
